# Ground penetrating radar at work: A realistic perspective on utility surveying in the Netherlands through a comprehensive ground-truth dataset

**DOI:** 10.1016/j.dib.2024.110329

**Published:** 2024-03-16

**Authors:** Ramon B.A. ter Huurne, Léon L. Olde Scholtenhuis, André G. Dorée

**Affiliations:** Department of Construction Management and Engineering, Faculty of Engineering Technology, University of Twente, PO BOX 217, 7500 AE, Enschede, the Netherlands

**Keywords:** Construction, Ground penetrating radar, Ground-truth, Practice, Utility surveying

## Abstract

This dataset provides a comprehensive compilation of Ground Penetrating Radar (GPR) surveys across 125 utility surveying activities in the Netherlands. The dataset details the specific use of GPR in each authentic real-life utility surveying activity, whether employed independently or as a complementary tool alongside existing surveying methods, with or without post-processing. The dataset includes 959 radargrams, ground-truth information obtained from trial trenches, and an inventory of construction, geophysical, infrastructural, and technical features. The GPR utilised in all activities is an air-coupled radar with a 500 MHz frequency antenna, a GNSS RTK positioning system, and a measuring wheel encoder. This ground-truth dataset provides researchers with a valuable resource to further assess the practical efficacy of GPR as a utility surveying method, refine radargram processing algorithms and techniques, and explore the possibilities of predictive modelling.

Specifications Table

**Table:** 

Subject	Civil and Structural Engineering
Specific subject area	A Realistic Perspective on Utility Surveying with Ground Penetrating Radar
Type of data	Raw radargrams in SEG-Y file format (.sgy). Processed images of survey line maps per activity in .png. Processed images of cross-sections of trial trenches in .png. Filtered metadata table describing the type of GPR application and site characteristics in .csv (one general file for all activities). Processed codebook table of metadata file in .pdf.
Data collection	Radargrams (.sgy) were collected using an air-coupled Ground Penetrating Radar with a 500 MHz frequency antenna, a GNSS RTK receiver, and a measuring wheel encoder. A tablet using proprietary software was used to operate the GPR and visualise the radargrams. Ground-truth data (.png) were collected through trial trenching using analogue and georeferenced measuring equipment. Metadata of the site (.csv) were collected through interviews, observations and conversations with the workers. The ground relative permittivity was calculated using the velocity of GPR waves determined through the hyperbola fit function in Reflex-W software (version 9.1.3).
Data source location	Data were collected across thirteen construction projects in the Netherlands located in or around Enschede, Eindhoven, Arnhem, Zwolle, Helmond, Helvoirt, Berkel-Enschot, Rotterdam, Zaandam, Oudewater and Feanwalden.
Data accessibility	Repository name: Ground Penetrating Radar dataset with ground-truth data of utility surveying activities Direct URL: https://data.4tu.nl/datasets/96303227-5886-41c9-8607-70fdd2cfe7c1 DOI: https://doi.org/10.4121/96303227-5886-41c9–8607-70fdd2cfe7c1.v1

## 1 Value of the Data

• The intrinsic value of this dataset lies in its real-world origins. Unlike controlled or laboratory-based settings, this dataset is derived from authentic utility surveying activities. As such, it encapsulates the intricate complexities that subsurface utilities present in authentic scenarios.
• Encompassing 125 utility surveying activities, the dataset brings together information on how GPR was applied, a vast array of GPR radargrams — totalling 959 — and accompanying trial trench (ground-truth) data. This rich collection encapsulates an expansive set of utility surveying scenarios.
• The dataset's ground-truth foundation presents a unique opportunity for technology assessment experts to evaluate the capabilities of GPR across an expansive array of authentic surveying conditions. Researchers can utilise this data to explore the practical value of GPR as a utility detection technology in the construction domain, aiding in identifying its use cases in realistic contexts of work.
• The raw radargrams in the dataset serve as a valuable resource for assessing and refining radargram processing algorithms and techniques. The diverse range of utility diameters, materials, and intricate complexities present in the dataset provides a dynamic testing ground. This testing environment allows researchers to scrutinise the efficacy of processing algorithms and determine optimal pathways for their evolution.
• By leveraging the dataset's information on the type of GPR deployment for each of the 125 utility surveying activities, researchers can delve into the development of predictive models that anticipate the applicability of GPR in forthcoming surveying activities. Such predictive models empower workers with valuable insights into GPR's anticipated value, enhancing the effectiveness of its onsite deployment.

## 2 Background

As construction projects increasingly involve works with or adjacent to subsurface utilities, the demand for accurate and comprehensive information regarding their locations and attributes becomes critical. This need stems from the ongoing growth and urbanisation of societies, advancements in communication technologies, and the active pursuit of long-term agendas such as energy transition and climate adaptation [1]. Organisations preparing for construction works rely heavily on obtaining this information, as failure to do so can result in utility strikes; a significant issue within the sector, demonstrated by the nearly 47 thousand reported instances in the Netherlands alone in 2022 [2]. Existing literature advocates for adopting Ground Penetrating Radar (GPR) as a geophysical detection technology to assist construction organisations in better utility detection [3].

GPR is a geophysical method offering a non-intrusive and rapid means of utility surveying [4]. This technology operates by transmitting electromagnetic signals into the subsurface, where variations in the electric and dielectric properties of the medium cause the signal to disperse and reflect back to the GPR receiver. These reflections, typically manifesting as hyperbolic shapes for utilities, serve as the foundation for generating a ‘radargram’. Through analysis of this radargram, utility depth and, to a lesser extent, dimensions and material composition can be deduced. While the radar is always considered to provide the ‘right’ information, it remains essential to interpret its outcomes and determine how to use them in a practical work context [4]. Therefore, ongoing research focuses significantly on GPR's utility detection capabilities with an emphasis on optimising radargram processing [5,6] and exploring innovative and experimental (3D) scanning techniques [7,8].

However, the majority of existing research on GPR is conducted within controlled laboratory settings, limiting its generalizability to the complexities and uncertainties encountered in real-world applications. Real-world scenarios present challenges such as non-homogeneous subsurface mediums, closely packed utilities installed in non-linear patterns, uncertainty in utility locations, and the context-specific surveying requirements of construction organisations. Consequently, there often exists a disparity between the outcomes of laboratory-based studies and construction organisations' anticipated value of GPR. This disparity has led to the frequent ‘failure’ of GPR applications [3,9], as construction teams’ surveying requirements could not be adequately addressed. Consequently, there is a noticeable lack of consideration for GPR in surveying practices within the construction industry. A realistic assessment of the value of GPR in authentic utility surveying scenarios is, therefore, necessary to expedite its adoption in the construction context.

This article provides an empirically rich dataset derived from applying GPR in real-life utility surveying activities. The value of this dataset lies in its ability to provide researchers with empirical data that encapsulates the intricate complexities of real-life utility surveying scenarios. This data can be used to (1) evaluate the practical capabilities of GPR as a detection technology across an expansive array of utility surveying conditions, (2) assess and refine radargram processing algorithms and techniques, and (3) train and develop predictive machine-learning driven models that anticipate the applicability of GPR in forthcoming surveying activities.

## 3 Data Description

**Fig. 1:**
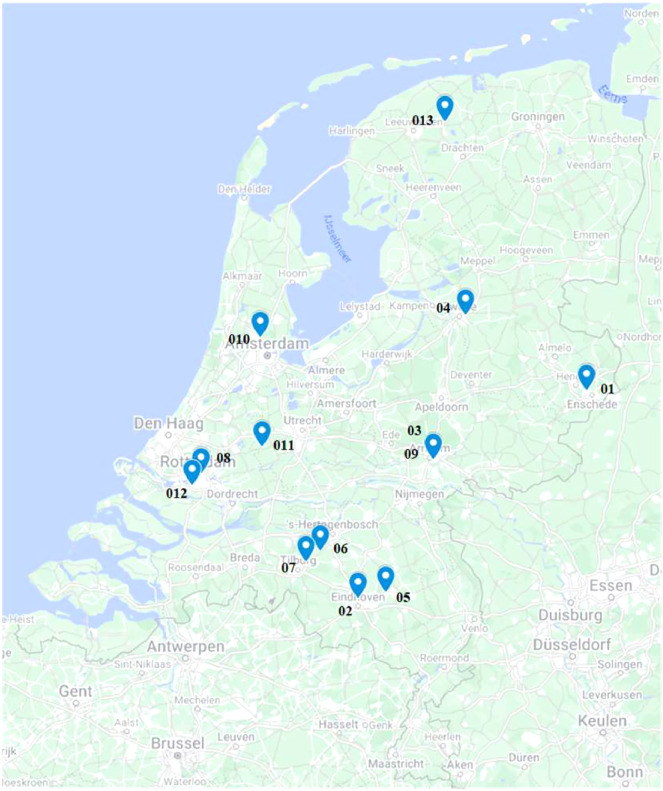
Map of project locations spread across the Netherlands.

This article outlines a dataset encompassing 125 utility surveying activities conducted across thirteen construction projects in the Netherlands between April 2020 and March 2021 [10]. These projects were situated in or around various Dutch cities and towns, including Enschede, Eindhoven, Arnhem, Zwolle, Helmond, Helvoirt, Berkel-Enschot, Rotterdam, Zaandam, Oudewater and Feanwalden (Fig. 1). The projects in the dataset are identified numerically from one to thirteen. More data may be added to the dataset in the future.

**Fig. 2:**
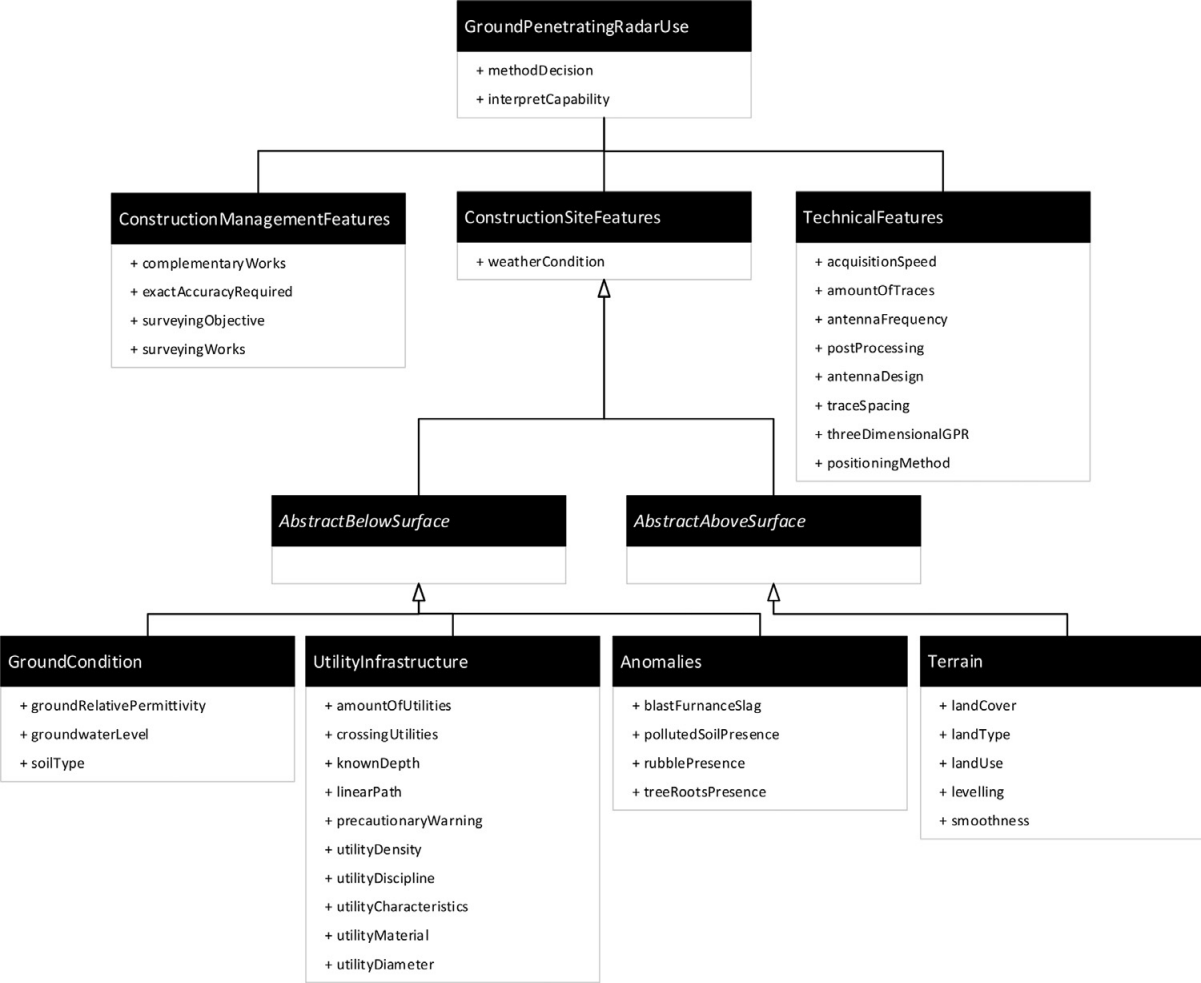
Taxonomy of the dataset.

The dataset includes filtered metadata for each surveying activity, describing how a Ground Penetrating Radar (GPR) was applied and under what conditions the activity occurred. These condition features are grouped into three categories: construction management-, construction site-, and technical-related features. The construction site-related features are divided into below-surface features (i.e., ground condition, utility infrastructure, and anomalies) and above-surface features (i.e., terrain type and surroundings). Fig. 2 provides an overview of the taxonomy of the metadata. The metadata for all surveying activities is captured in a .csv file. A codebook, which details each feature, its attributes, and its values, is also enclosed in the dataset.

The primary focus of the dataset revolves around detailing the application of GPR in utility surveying activities. The dataset differentiates among three types of GPR methods: the standalone method of GPR with post-processing of radargrams (referred to as ‘0’ in the dataset), the standalone method of GPR without post-processing of radargrams (referred to as ‘1’), and the complementary method of GPR alongside trial trench verification (referred to as ‘2’). Both standalone methods denote the use of GPR as an independent surveying technique capable of meeting the specific surveying requirements of the activity. Depending on whether post-processing is necessary in a given case, the dataset distinguishes between these two as components of the standalone application of GPR. In instances where GPR alone could not meet the surveying requirements of the activity, it was employed as a complementary method alongside trial trenching. The choice of method was guided by the expertise of the GPR operator and thes proficiency in interpreting radargrams. Throughout all activities, the same GPR operator, who demonstrated a high level of skill in both the operational and interpretative aspects of GPR usage, was involved.

The dataset provides information about the conditions governing the application of the specific GPR method. The construction management-related features outline utility surveying objectives, planned construction works, accuracy requirements, and additional construction activities. Most surveying activities in the dataset were geared towards verifying existing utility maps, frequently together with utility replacement or installation works. Construction organisations commonly did not mandate pinpoint accuracy in determining utility locations.

The construction site-related features are described through both below-surface and above-surface features, complemented by the general condition of the weather. Below-surface features provide insight into the ground conditions within the surveying area, the existing utility infrastructure, and the identification of anomalies. Specifically, ground conditions detail the soil's relative permittivity, the relative groundwater level compared to utility depth, and the soil type. The dataset predominantly focuses on urban areas with sandy ground conditions, with a few instances involving clayey soil types. Notably, most utilities were situated above the groundwater level.

The infrastructural features describe the utilities as found on site. This includes the amount of utilities and their respective disciplines, materials, and diameters. Additionally, it notes whether there was an elevated risk of utility strikes, if the depth of the utilities was known, whether utilities were crossing, and the orientation of their paths (linear or curved). At the surveyed areas within the dataset, a minimum of 2 utilities and a maximum of 23 utilities were identified. Utility disciplines encompass water, electricity, oil/gas/chemicals, sewage, and telecommunications, with diameters ranging from 16 mm to 1326 mm. Some activities were flagged with an increased risk of utility strikes. Most utilities followed a linear orientation. The dataset includes notes for specific utility conditions, such as being shielded with a cover, being bundled, featuring a diameter or material transition, or being installed in a conduit (a larger pipe designed to protect inner utilities).

The dataset also specifies the presence of anomalies in the subsoil. Four types of anomalies were considered, namely the existence of blast furnace slag, polluted soil, rubble, and tree roots. Across the dataset, anomalies were found to a limited extent, with rubble being the most frequently observed among the four types.

The above-surface features provide insights into the type of terrain and environment where the surveying activity occurred. Specifically, the terrain feature outlines the land cover, type, and use, along with the levelling and smoothness of the terrain. While most surveying occurred on paved surfaces such as sidewalks, streets, and parking areas, unsurfaced surfaces like greenery were also present.

The technical-related features outline the operational and technical details concerning the application of GPR. Specifically, they describe the acquisition speed of GPR data collection, the number of traces collected and their spacing, the GPR antenna design type and its frequency, the employed positioning method for utility location determination, whether post-processing of radargrams was conducted, and if the GPR facilitated the collection of three-dimensional data. The same GPR equipment was consistently used across all surveying activities, as further elaborated in the design, materials, and methods section.

**Fig. 3:**
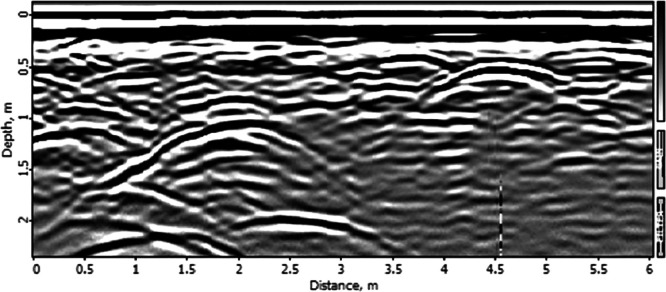
Example output of a radargram (.sgy).

**Fig. 4:**
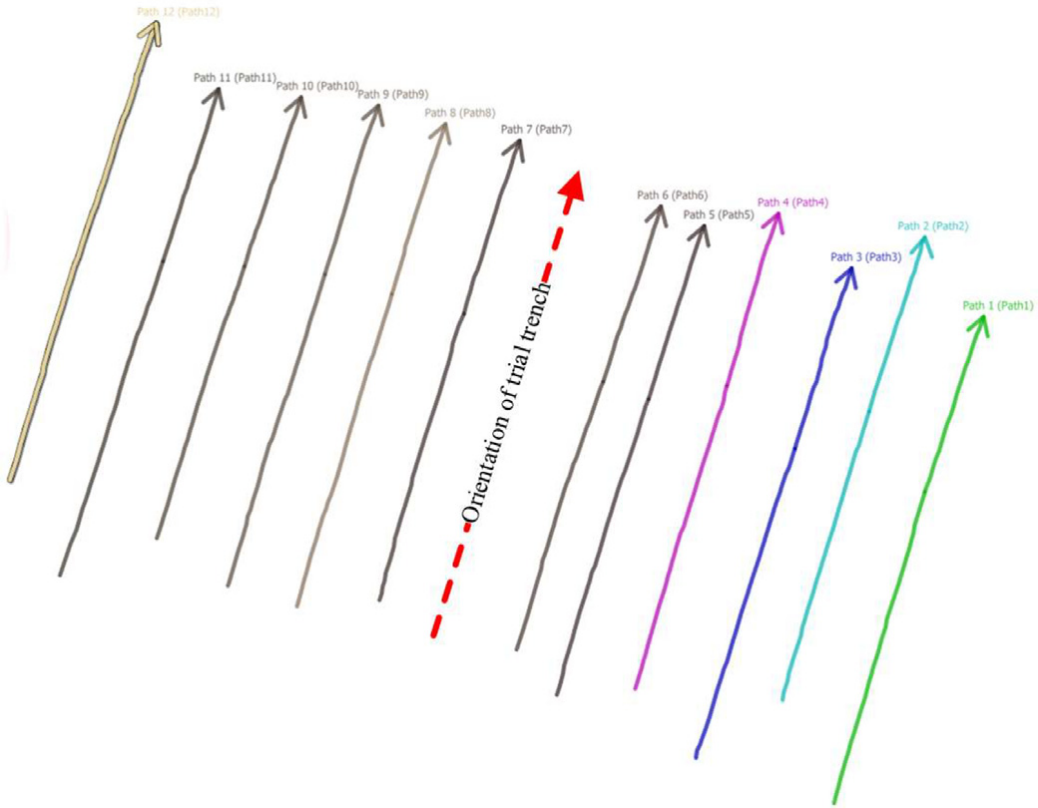
Map of radargrams including orientation of trial trench (.png).

Alongside the filtered metadata, the dataset includes the surveying data itself. These were gathered through Ground Penetrating Radar (GPR) and trial trenching methodologies. The data repository offers raw and georeferenced radargrams and an overview of processed ground-truth data per surveying activity. Radargram counts per activity range from 2 to 26, culminating in 959 radargrams for the entire dataset. Fig. 3 presents one of the included radargrams. Additionally, for each activity, survey lines are visualised on a map together with the orientation of the trial trench, as seen in Fig. 4.

**Fig. 5:**
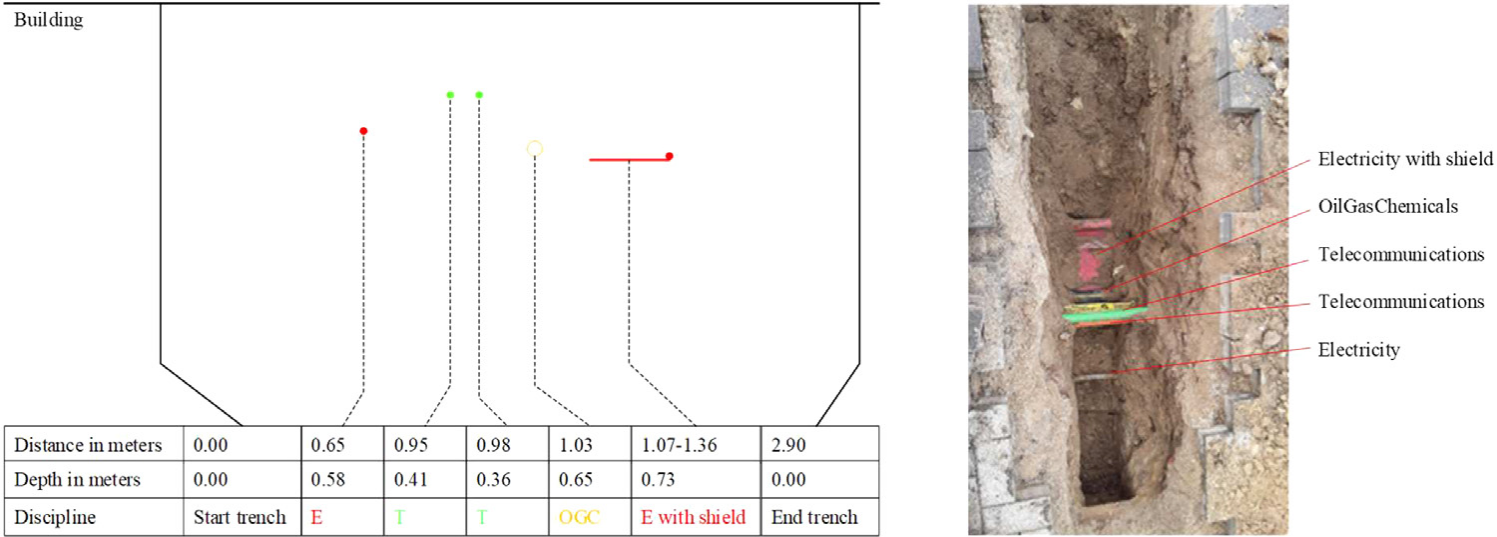
Example output of ground-truth data through a cross-section and captured images of the trench (.png).

Each activity comes with corresponding ground-truth data collected through trial trenching. The processed ground-truth data provides cross-sections of the trenches detailing utility location and their type, captured images of the exposed utilities, or a combination of these. Fig. 5 provides an example of how ground-truth data for one of the activities is depicted within the dataset.

Notably, the ground-truth data lacks georeferencing due to confidentiality constraints. Geospatial information has been omitted to preserve data and utility location confidentiality. The radargrams, however, are georeferenced.

## 4 Experimental Design, Materials and Methods

Data were collected through a three-stage process: collecting metadata to describe the surveying conditions, collecting radargrams, and collecting ground-truth data. The following sections describe these stages' experimental design, materials, and methods.

### 4.1 Metadata

Metadata to describe the surveying conditions was captured through a combination of exploratory interviews and field observations. Before surveying, we organised interviews with one or two key actors from each construction project. These interviews, lasting approximately one hour, involved supervisors, project managers, and project clients. We asked them to explain their utility surveying objectives, expected outcomes, planned construction works, and distinctive characteristics of the surveying locations. Additionally, we acquired utility maps from these organisations, sourced through the Dutch national and regulated utility-data exchange platform [11]. These maps provided insights into the number and types of utilities present in the surveying areas.

Next, onsite surveying conditions were gathered. We compiled field notes through direct observations of the surveying areas, guided by insights from previous GPR studies [6,^8^,9]. These studies emphasised how soil types, groundwater levels, surface characteristics, and subsurface anomalies influence GPR output. The soil type observations were conducted after construction organisations excavated trenches. This allowed for a visual inspection of the type of soil. The ground relative permittivity values in the dataset were calculated after the observations. The velocity of GPR waves through the soil ($\upsilon_{m})\;$was determined using the Reflex-W software's (version 9.1.3) hyperbola fit function. Using this velocity ($\upsilon_{m})$ and the speed of light (c), we calculated the ground relative permittivity ($\varepsilon_{r}$) through Eq. (1).

(1) $$\varepsilon_{r}={\left(\frac{c}{\upsilon_{m}}\right)}^{2}$$

Following the GPR surveying onsite, the GPR operator participated in discussions with the project teams, integrating his insights from the surveys with the construction expertise of the teams. Collectively, they determined the most suitable GPR method for achieving the specific surveying objectives at the construction site. This collaborative process resulted in a GPR method decision (i.e., standalone with post-processing, standalone without post-processing, or complementary alongside trenches) for each activity documented in the dataset.

Qualitative coding disseminated the interview and field note data towards the filtered metadata features. The principles of Corbin and Strauss [12] guided this process. First, open coding was used to code the data line-by-line. Examples of codes include ‘replacement of utilities’ and ‘survey on the sidewalk’. Subsequently, axial coding was applied to link and group these codes into broader categories. These categories collectively constitute the features encapsulated in our metadata.

### 4.2 Radargrams

We employed an air-launched GPR featuring a 500 MHz antenna complemented by Spectre's SP80 GNSS (Global Navigation Satellite System) RTK (Real-Time Kinematic Positioning) receiver. This combination enabled the recording of subsurface objects' geodetic locations in the x, y, and z axes. The air-launched design of the GPR resulted in the antenna being positioned just a few centimetres above the surface. This characteristic is visually evident within the radargrams, where the 'airgap' effect is discernible. In addition, the GPR was equipped with a measuring wheel encoder mechanism, enabling data transmission solely when the wheels were set in motion. The GPR used did not facilitate the collection of three-dimensional data.

**Fig. 6:**
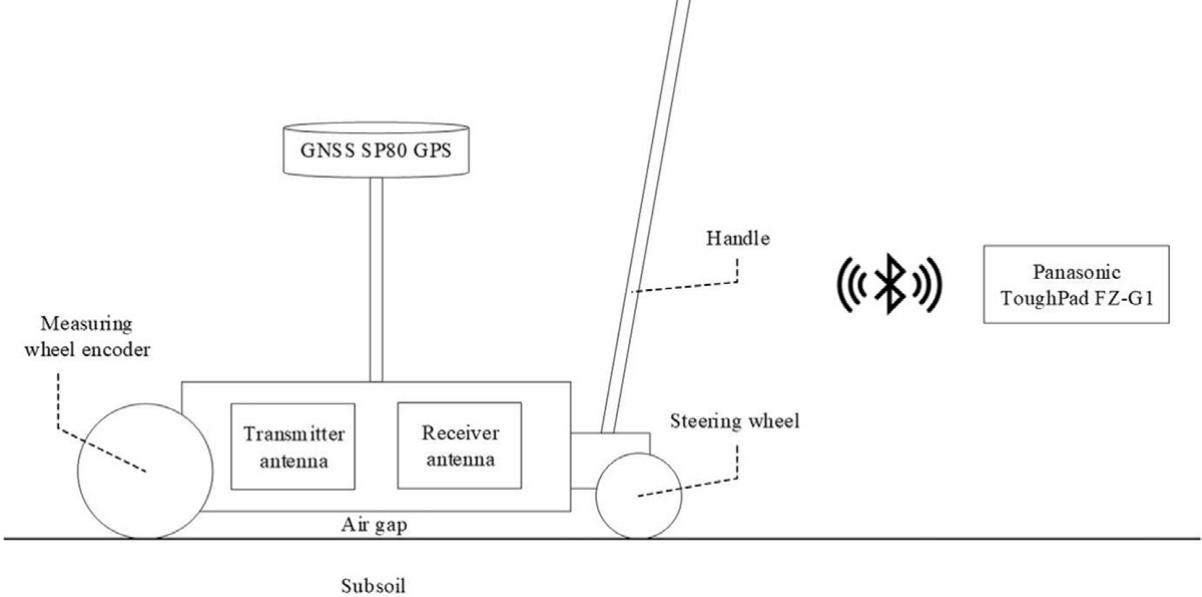
Schematic configuration of the GPR experimental setup.

Our GPR survey approach maintained a trace spacing of 0.02 m, ensuring fine granularity. Per trace, 512 samples were recorded using a 50 ns time range. To manage and control the GPR system, we used a Panasonic ToughPad FZ-G1, which utilised proprietary software tailored to our GPR model. This tablet was the control hub, communicating with the GPR device via Bluetooth. A visual depiction of the experimental setup is presented in Fig. 6.

**Fig. 7:**
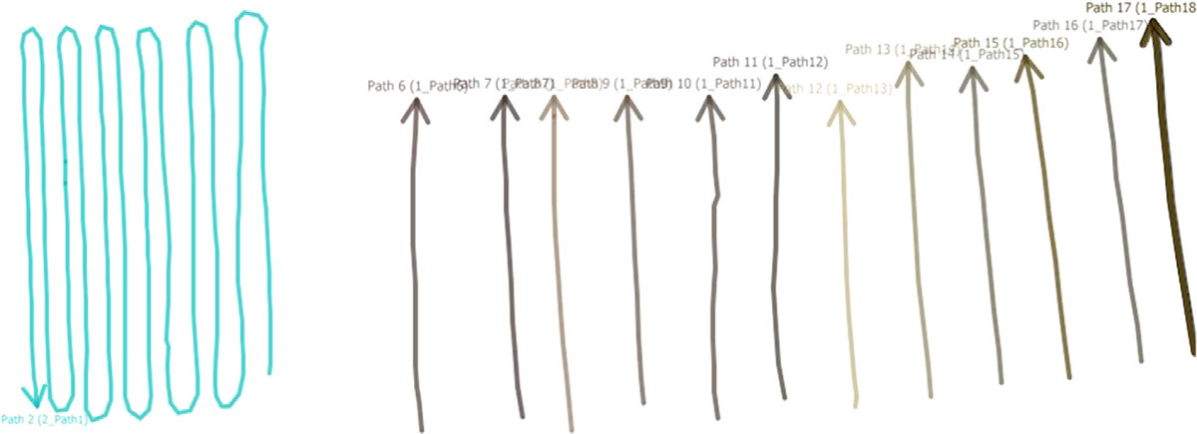
Continuous survey line (left side) and separate survey lines (right side).

GPR measurements were conducted at every location where the construction organisations had planned a trial trench. At these locations, survey lines were oriented perpendicular to the utilities. The emergence of a hyperbola in the radargram signified the crossing of a utility. Multiple survey lines were walked for each surveying activity to distinguish utility lines from potential anomalies. The range of survey lines varied from 2 to 26, generally spaced 1 meter apart – a suitable interval up to a busy urban setting [13]. Survey lines were either separately collected or as one continuous trace in a ‘zigzag’ pattern depending on the available space to manoeuvre the GPR device. Examples of these two approaches are presented in Fig. 7.

The researcher walked the GPR along these survey lines to collect the radargrams. Data were hence collected at walking speeds. Employing the GNSS RTK receiver of the GPR, we could visualise the maps of the survey lines for each activity. However, in some surveying activities, tall buildings obstructed the GNSS signal. As a result, some measurements have inaccurate or missing mappings of the survey lines.

The radargrams were collected in the SEG-Y format. This is an open standard established by the Society of Exploration Geophysicists in 1975 [14]. The format is the recommended archival file format for GPR data [15]. The SEG-Y files in the dataset are unprocessed and in their raw state. They are directly imported from the GPR device.

### 4.3 Ground-truth

Ground-truth data were obtained through the excavation of trial trenches. The construction organisations themselves undertook this task. The digging process encompassed both manual and mechanical techniques. Guided by the utility maps at their disposal, workers dug these trial trenches to verify the utilities represented on the maps and pinpoint specific utilities or free (unoccupied) subsoil areas.

**Fig. 8:**
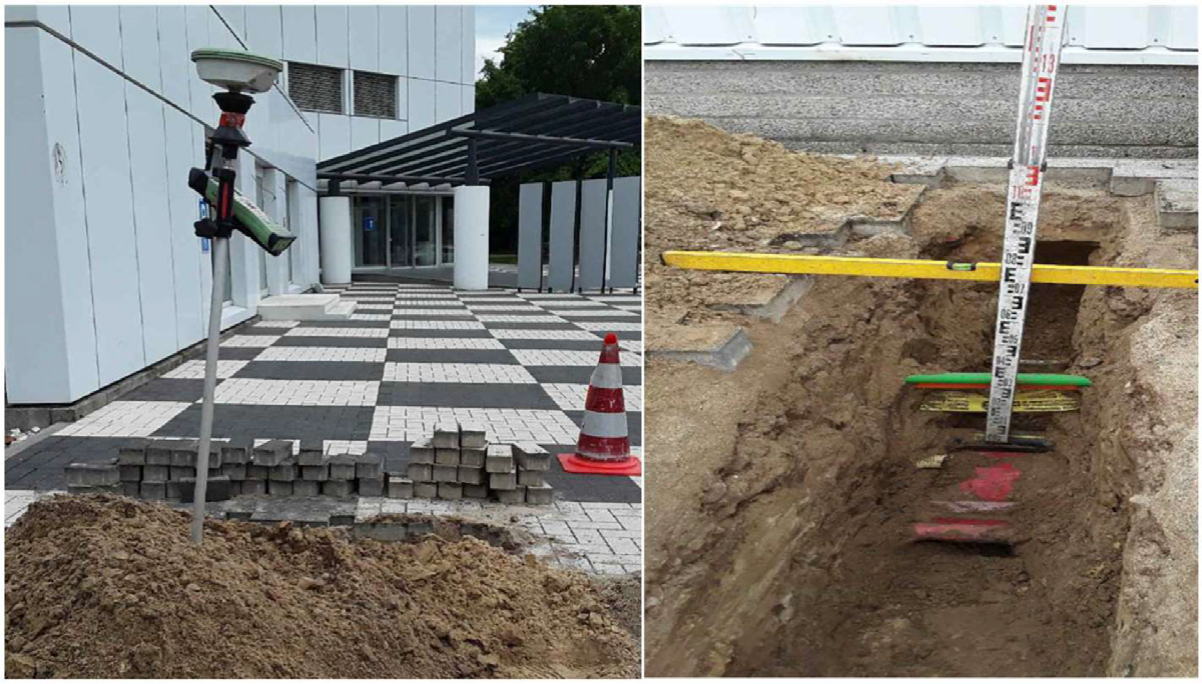
Use of digital GNSS technology (left side) and analogue measures (right side) to determine utility locations.

After the trenches were excavated, utility locations were determined. This process involved analogue methods, including tape measures and water levels, or digital methods using GNSS technology. Analogue approaches entailed recording the relative location and depth of utilities, while the GNSS technology facilitated the collection of geodetic coordinates in the x, y, and z axes. The construction organisations carried out or arranged the measurement procedures, with the added collection of utility discipline, material, and diameter types. Photographs were taken before the trenches were sealed. The visual depiction of a utility location recording, as witnessed in the surveying activities, can be found in Fig. 8.

Following the measurements, we acquired either cross-sectional data from the trial trenches or georeferenced CAD files supplied by the construction organisations. However, these CAD files are not enclosed in this dataset due to confidentiality constraints. Instead, the dataset contains cross sections or images of the exposed utilities, or a combination of these.

## 5 Limitations

The GPR and ground-truth dataset presents three limitations. First, several SEG-Y files within the dataset lack georeferencing information. While a GNSS RTK receiver was utilised for each GPR measurement, GNSS signal obstruction led to measurements only using the measuring wheel encoder. As a result, these SEG-Y files lack the geospatial context for the measurements.

Second, certain instances within the dataset feature GPR measurements in rough terrains, for example, ditches alongside roads. Such uneven and demanding topography led to instances where the measuring wheels could not maintain consistent ground contact. This compromised both the data throughput and its overall quality. Extracting information about subsurface utilities from the SEG-Y files in these cases becomes more challenging.

Third, the material and diameter type are not included for every utility in the dataset. Various construction organisations managed the collection of ground truth data, each adopting distinct approaches. This divergence resulted in instances where material and diameter details were omitted. In such situations, our ability to personally inspect the trial trench to collect this information was also limited, as trenches often had already been sealed due to safety considerations.

## Ethics Statement

The present work did not involve the use of human subjects, animal experiments, or data collected from social media platforms.

## CRediT authorship contribution statement

**Ramon B.A. ter Huurne:** Conceptualization, Methodology, Software, Investigation, Data curation, Writing – original draft. **Léon L. Olde Scholtenhuis:** Conceptualization, Supervision, Writing – review & editing. **André G. Dorée:** Conceptualization, Supervision, Writing – review & editing.

## Data Availability

Ground Penetrating Radar dataset with ground-truth data of utility surveying activities (Original data) (4TU.ResearchData).
